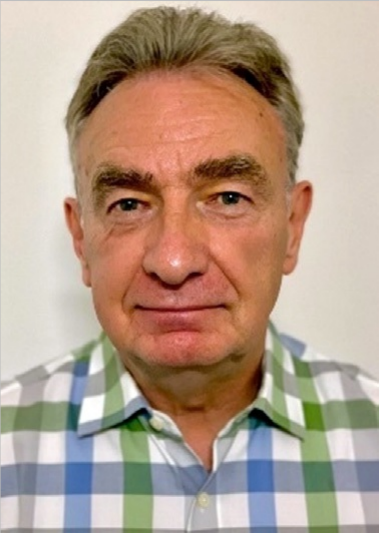# Alkali‐Metal Mediation: Diversity of Applications in Main‐Group Organometallic Chemistry

**DOI:** 10.1002/anie.202010963

**Published:** 2020-12-03

**Authors:** Thomas X. Gentner, Robert E. Mulvey

**Affiliations:** ^1^ Department of Pure and Applied Chemistry University of Strathclyde Glasgow G1 1XL UK

**Keywords:** alkali metals, catalysis, metallation, organolithium compounds, π–arene interactions

## Abstract

Organolithium compounds have been at the forefront of synthetic chemistry for over a century, as they mediate the synthesis of myriads of compounds that are utilised worldwide in academic and industrial settings. For that reason, lithium has always been the most important alkali metal in organometallic chemistry. Today, that importance is being seriously challenged by sodium and potassium, as the alkali‐metal mediation of organic reactions in general has started branching off in several new directions. Recent examples covering main‐group homogeneous catalysis, stoichiometric organic synthesis, low‐valent main‐group metal chemistry, polymerization, and green chemistry are showcased in this Review. Since alkali‐metal compounds are often not the end products of these applications, their roles are rarely given top billing. Thus, this Review has been written to alert the community to this rising unifying phenomenon of “alkali‐metal mediation”.

## Graphical abstract

Alkali metals top the league when it comes to mediating reactions. Organolithium compounds have long been their captain, but now organosodium and organopotassium compounds are finding their form too. This Review highlights their victories in homogeneous catalysis, organic synthesis, low‐valent aluminium chemistry, polymerization, and green chemistry.

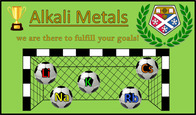

## 1 Introduction

Pioneered by Wilhelm Schlenk and Joanna Holtz in 1917,^[1]^ organolithium compounds have made a phenomenal contribution to the development of chemistry across the whole landscape of the periodic table. They are masters of a diverse range of metal‐mediated reactions, for example, operating as bases in metallation reactions, as nucleophiles in addition reactions to unsaturated molecules, as metal–halide exchange agents in metathesis reactions with organic halides, as ligand‐transfer agents in transmetallation reactions with main‐group, transition‐metal, and lanthanide/actinide compounds, or as cross‐coupling partners in Pd catalysis.^[2]^ The mediation tag stems from the action of the organolithium intermediates in these reactions, which make possible onward reactivity towards the desired organic products or organometallic compounds of other metals. By contrast, the other common alkali metals sodium and potassium have contributed to a much lesser degree to this conventional alkali‐metal mediation chemistry, although, interestingly, Wanklyn started investigations on organosodium and organopotassium compounds almost half a century before organolithium reagents revolutionized organometallic chemistry. However, the general lower stabilities and lower solubilities of these compounds complicated their application.[^2m^, ^2n^, ^2o^, ^2p^]

Interestingly, however, new unconventional alkali‐metal‐mediated applications are emerging where this organolithium dominance is often challenged. At present, it is a complicated sporadic picture, as these new applications cut across different themes including main‐group homogeneous catalysis, stoichiometric organic synthesis, low‐valent main‐group metal chemistry, polymerization, and green chemistry. Systematic studies spanning the whole of Group 1 (Li–Cs) are still relatively rare, but there are some studies where alkali‐metal effects are clearly at play, that is, there are gradations of mediation efficiency depending on the identity of the alkali metal. Remarkably, in a high proportion of examples, potassium appears to be outperforming its smaller sibling lithium. What follows is a critical selection of snapshots, mostly from the past five years, that spotlight the importance and increasing prominence of this new, often inverted, alkali‐metal mediation efficiency. It is written with the intention of promoting alkali‐metal mediation as a unifying concept of tremendous scope, with the aim of attracting other researchers, especially early career chemists looking for exciting new research lines, who can take the subject to even greater heights.

## 2 Emerging Alkali‐Metal‐Mediated Applications

### 2.1 Alkali Metals in Homogeneous Catalysis

**Scheme 1:**
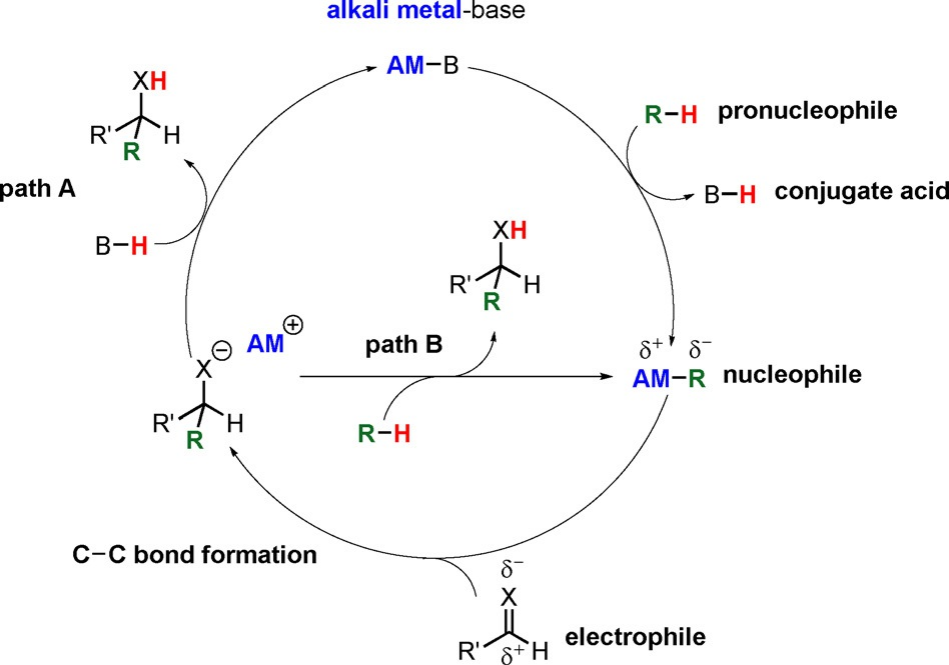
General mechanism for Brønsted base catalysed C−C addition reactions.

Since the beginning of the 21st century, there has been an increasing interest in common alkali metal (Li, Na, K) based catalysts and the reasons are clear. These include the low toxicity and costs compared to those of transition metals and lanthanides, and the availability everywhere on the Earth's crust and in the water of the oceans. These factors make them attractive and sustainable elements for potential use in a multitude of transformations even though their redox chemistry and the number of oxidation states are limited. A growing number of reports are appearing where the metals of the first main group are used in catalytic applications, for example, hydroboration,^[3]^ intermolecular and intramolecular hydroamination,^[4]^ hydrophosphination/hydrophosphorylation,^[5]^ hydrosilylation,^[6]^ hydrogenation,^[7]^ dehydrogenation (or dehydrocoupling),^[8]^ and Brønsted base catalysed C−C additions.^[9]^ For the last application, first reports date back to the 1950s from Pine and Wunderlich, who described the addition of alkylbenzenes to styrenes with catalytic amounts of Na or K. However, because of the high reactivity of the pure metals, only low selectivities were obtained.^[9t]^ Today, there are many examples where an alkali metal catalytically converts cheap feedstock compounds, such as aldehydes, amides, arenes, nitriles, imines, or allyl derivatives, into more value added compounds. The catalysts show good selectivity in general, and even asymmetric additions are reported.[^9n^, ^9p^, ^9q^, ^9r^] Scheme 1 presents a general mechanism for this C−C bond formation. The alkali‐metal base (AM−B) deprotonates the pronucleophile (R−H) to form the reactive nucleophile (AM−R) under liberation of the conjugate acid (B−H). The high electropositivity of the alkali metal endows the AM−R bond with strong polarity and thus installs a high negative charge on the carbanion. The nucleophile (AM−R) can then react at the partial positively charged centre of the electrophile to induce the C−C bond formation. Typically, the resulting intermediate is a powerful base that can form the desired product by deprotonation of either the conjugate acid (B−H; path A) or the pronucleophile (R−H; path B). In general, the latter species will be deprotonated when its acidic hydrogen atom has a lower p*K*_a_ value than that of the conjugate base.^[9j]^

**Scheme 2:**
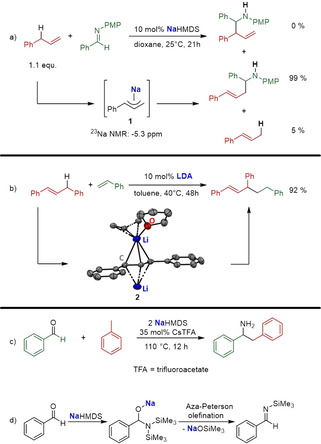
C−C bond formation between a) an imine and an allyl compound catalysed by NaHMDS; b) styrene and an allyl compound catalysed by LDA; c) an aldehyde and toluene by activation of the benzylic group through cation–π interaction; d) conversion of benzaldehyde into *N*‐(trimethylsilyl)benzaldimine by NaHMDS.

Since alkali‐metal bases are among the strongest bases, pronucleophiles with p*K*_a_ values higher than 35 can be converted. Schneider and co‐workers reported the allylic C(sp^3^)−H bond activation of alkenes and their addition to imines. They discovered that NaHMDS (HMDS=1,1,1,3,3,3‐hexamethyldisilazide) already showed outstanding activity (Scheme 2 a) and selectivity at room temperature, while surprisingly other HMDS‐based complexes with various metals (Li, Mg, Ca, Sr, Sn, Cu, Ag, Zr, Ce, Eu, Gd) showed no activity or less activity and selectivity (e.g. with K; internal/external product ratio=23:44). The superior mediation efficiency of Na over both Li and K was explained in general terms by “a favourable mix of electronegativity, formal charge and ionic radius” which is necessary for this reaction. Schneider and co‐workers were also able to detect the intermediate nucleophile **1** by ^23^Na NMR spectroscopy (−5.3 ppm) and assigned it to be η^3^‐coordinated.^[9o]^ In 2018, the Guan group was able to isolate an intermediate nucleophile from an LDA (LDA=lithium diisopropylamide) catalysed allylic C−H bond alkylation with styrenes (Scheme 2 b). Compound **2** was prepared by deprotonation of 1,3‐diphenylpropene with in situ generated LDA, thereby giving the first structure of a π‐allylic Li compound. The cationic lithium centre is coordinated by one THF molecule and two diphenylallyl anions through η^3^‐coordinations, thereby resulting in a polymeric structure. It is noteworthy that the reaction of compound **2** with styrene results in the formation of styrene oligomers, which indicates that the diisopropylamine is crucial to prevent polymerization (Scheme 1, path A). Moreover, the larger DA congeners of Na and K were found to perform worse than their smaller sibling lithium, with yields of only 9 % and 11 %, respectively.^[9i]^

For the functionalization of the aromatic compound toluene and its derivatives, a method that is based on cation‐π interactions is often applied. Here, the smaller alkali metals are supported by their biggest (non‐radioactive) sibling caesium. Whereas common bases such as MHMDS (M=Li, Na, K) are not able to deprotonate toluene because of insufficient basicity (p*K*_a_≈43 of toluene in DMSO;^[10]^ p*K*_a_≈26 of HN(SiMe_3_)_2_ in THF^[11]^), the addition of caesium‐containing compounds (e.g. CsF, CsTFA [TFA=trifluoroacetate]) makes the reaction feasible.[^9c^, ^9e^, ^9j^]

**Figure 1:**
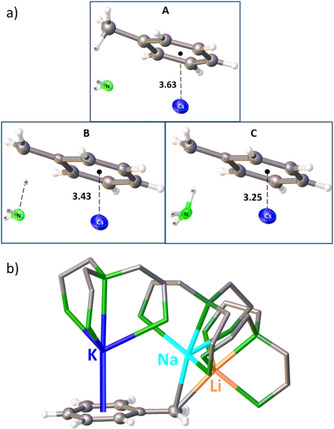
a) Calculated Cs–centroid distances (Å) for toluene upon benzylic C−H cleavage: reactant (A), transition state (B), product (C). b) Superimposition of the solid‐state structures of benzyl complexes of Li(orange), Na (turquoise), and K (blue). Methyl groups and all protons from the M_6_‐TREN ligand are omitted for clarity.

The large and soft caesium cation coordinates to the electron‐rich π‐system and slightly but significantly polarizes the aromatic system, thereby leading to a more acidic benzyl group. This reasoning was supported by a computational DFT study by Cundari and co‐workers, who calculated that the Cs^+^–centroid (toluene) distances decrease sequentially upon benzylic C−H cleavage along the reaction coordinate from 3.63 Å to 3.42 Å to 3.24 Å (neutral→transition state→product; see Figure 1 a). Moreover, they showed that decreasing the size of the metal is accompanied by a higher energetic barrier and that the cleavage is exergonic for all alkali metals except Li (*G* [kcal mol^−1^]: Li=3.9; Na=−0.3; K=−4.2; Rb=−4.3; Cs=−5.5).^[9s]^ The gradations in coordination behaviour of the alkali metals in benzylic systems was also shown by Robertson and co‐workers. They reported an eye‐catching series of Li, Na, and K benzyl complexes (Figure 1 b), which are broken down to monomers by the neutral, tetradentate Me_6_‐TREN ligand [Me_6_‐TREN=(Me_2_NCH_2_CH_2_)_3_N]. A nice trend is discernible from their crystal structures, namely that a larger metal coordinates more towards the π‐system of the aromatic ring rather than to the benzylic position.^[12]^

The concept of activation by cation‐π interaction has also been exploited by Walsh and co‐workers for the one‐pot aminobenzylation of aldehydes with toluene (Scheme 2 c). This study revealed that LiHMDS was unable to catalyse the reaction at all, whereas the corresponding Na (47 %) and K (35 %) amides showed moderate conversions. This trend is also consistent with the calculations from Cundari et al. alluded to above. The addition of 0.35 equivalents of CsTFA, the activator of the benzyl group, improved the reactivity remarkably for Li (94 %) and Na (95 %), in contrast to the modest mediation of K (50 %; here KHMDS is less efficient in the formation of the aldimine^[13]^). In this catalytic procedure the alkali‐metal base has two tasks: 1) It converts the aldehyde into aldimine through nucleophilic attack at the carbonyl carbon atom followed by an aza‐Petersen olefination (Scheme 2 d). 2) It deprotonates the toluene at the benzylic position to generate a nucleophile that is capable of attacking the previously formed aldimine. This one‐pot synthesis elaborates a variety of amines to a vast range of toluene derivatives through C−C addition of a broad range of aldehydes. Bearing in mind that the generated products can be used as convenient feedstocks for important bioactive building blocks, this simple one‐step procedure becomes even more attractive considering the use of cheap and non‐toxic alkali metals.^[9l]^

**Figure 2:**
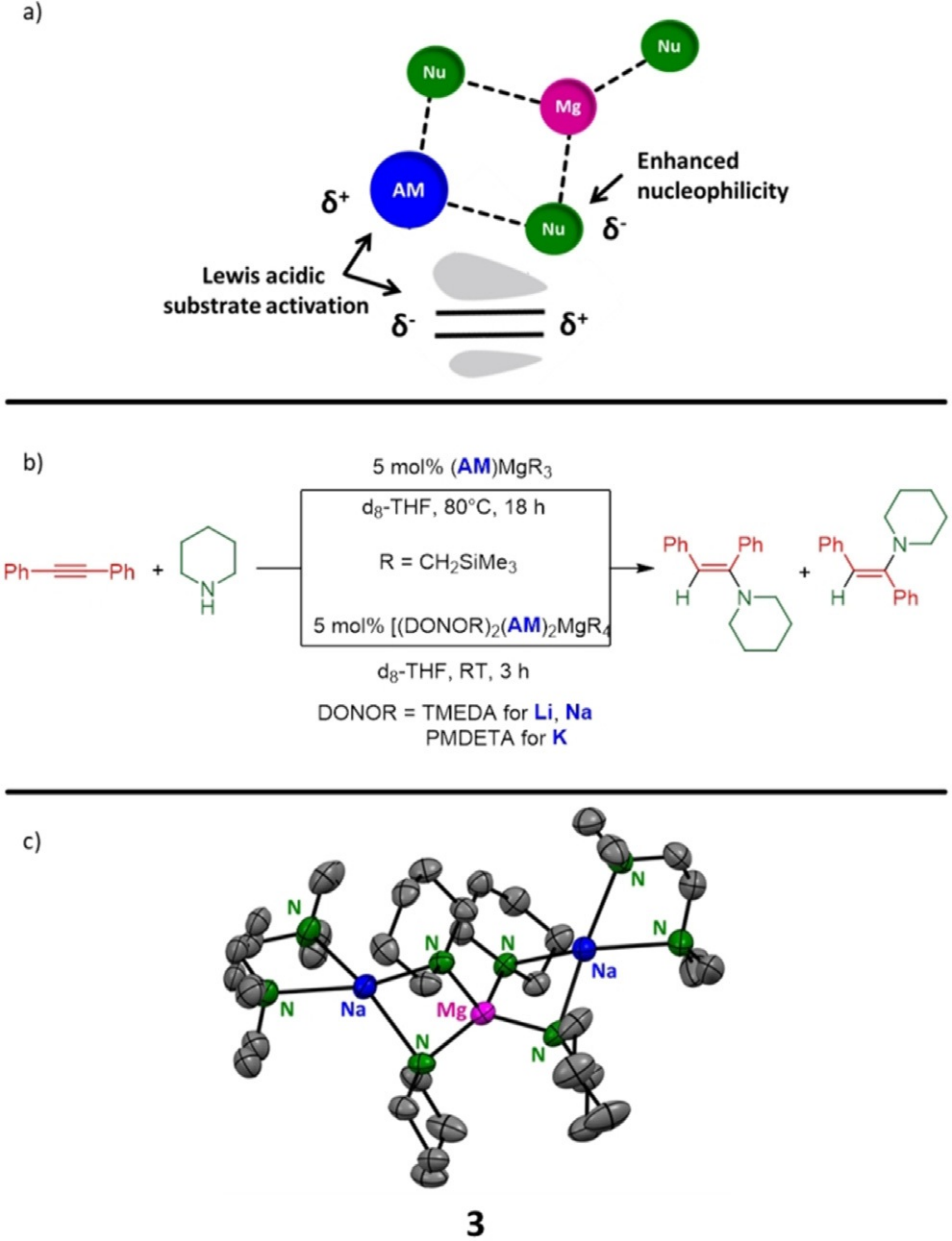
a) General illustration of the cooperativity between an alkali metal and Mg. b) Intermolecular hydroamination of diphenylacetylene with piperidine catalysed by (AM)MgR_3_ and (AM)_2_MgR_4_. c) Molecular structure of compound **3**.

Alkali metals do not only cooperate with each other, they show synergistic effects with metals from essentially the entire periodic table. For example, in 2019 Hevia and co‐workers reported a catalytic intermolecular hydroamination mediated by alkali‐metal magnesiates of the general forms (AM)MgR_3_ and (AM)_2_MgR_4_ (AM=Li, Na, K; R=CH_2_SiMe_3_).^[4a]^ Note that these complexes are referred to as lower order and higher order magnesiates, respectively, reflecting the relative stoichiometry of the R substituent. It is noteworthy that the catalytic activity of alkaline‐earth metals in intermolecular hydroamination decreases as the ionic radius decreases; thus, Mg shows poor reactivity for this transformation.^[14]^ However, this problem can be circumvented with the help of alkali metals, as depicted in Figure 2 a. The formation of an alkali‐metal magnesiate has two benefits: 1) enhanced reactivity of the nucleophile; and 2) substrate activation by polarization of the unsaturated bond by the alkali metal, which facilitates the nucleophilic attack and additionally brings the substrate in proximity to the Mg centre. This was evidenced by the Hevia group, who showed that on its own the magnesium alkyl compound Mg(CH_2_SiMe_3_)_2_ (80 °C, 24 h) could not catalyse the reaction between diphenylacetylene and piperidine, whereas lower order alkali‐metal magnesiates allow the conversion at 80 °C within 18 hours. Thereby, the reactivity increased from Li (48 %) to K (59 %) to Na (98 %) and the catalyst loading could be decreased to 2 mol %. A more significant boost in reactivity can be observed on utilizing higher order alkali‐metal magnesiates (AM)_2_Mg(CH_2_SiMe_3_)_4_, where the reaction already takes place at room temperature and is complete within 3 hours [reactivity: K(≥99 %)>Na(28 %)>Li(0 %)]. Saturation of the coordination sphere of K by the addition of 18‐crown‐6 resulted in a complete shutdown of the reactivity. This finding confirms the proposal that the alkali metal functions as a Lewis base and activates the unsaturated substrate by polarization. X‐ray structure determination and DOSY spectroscopy of the intermediate compound **3** (formed by the stoichiometric reaction between 4 equivalents of piperidine and [(TMEDA)_2_(Na)_2_Mg(CH_2_SiMe_3_)_4_]) additionally supports the hypothesis. As shown in Figure 2 c, compound **3** displays a contacted ion‐pair structure, which is crucial for effecting communication between the two metals. DOSY NMR measurements confirmed that this structure is present for lower order alkali‐metal magnesiates in solution.

**Scheme 3:**
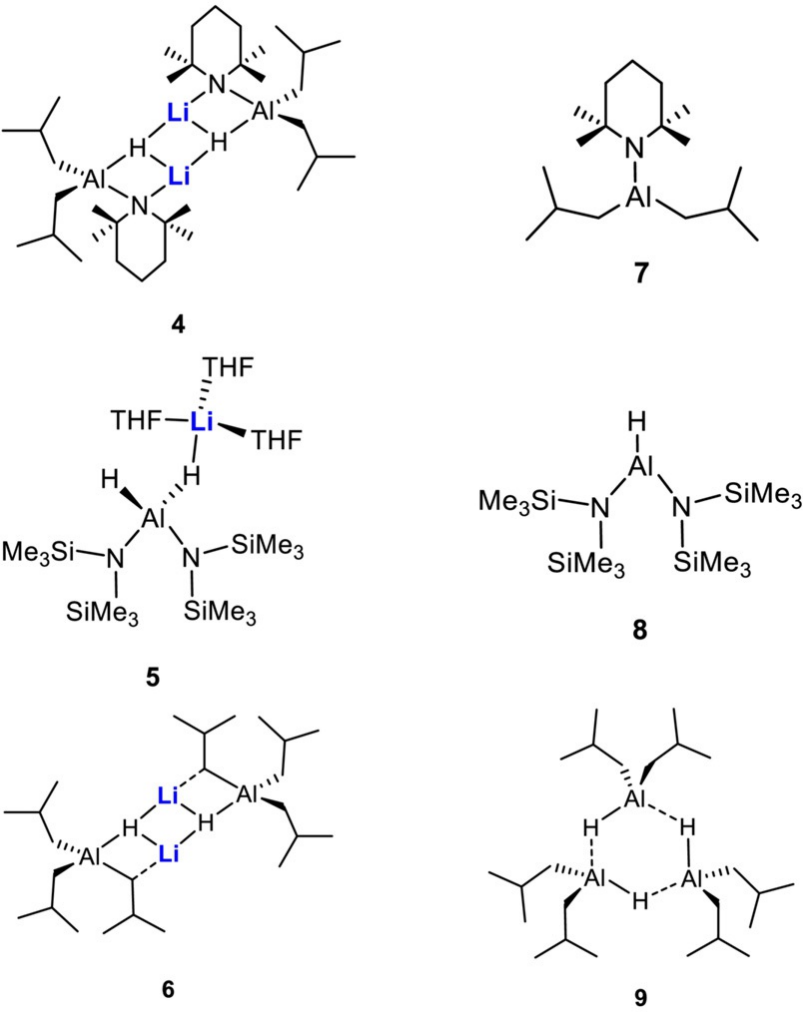
Hydroboration catalysts **4**–**9**.

**Scheme 4:**
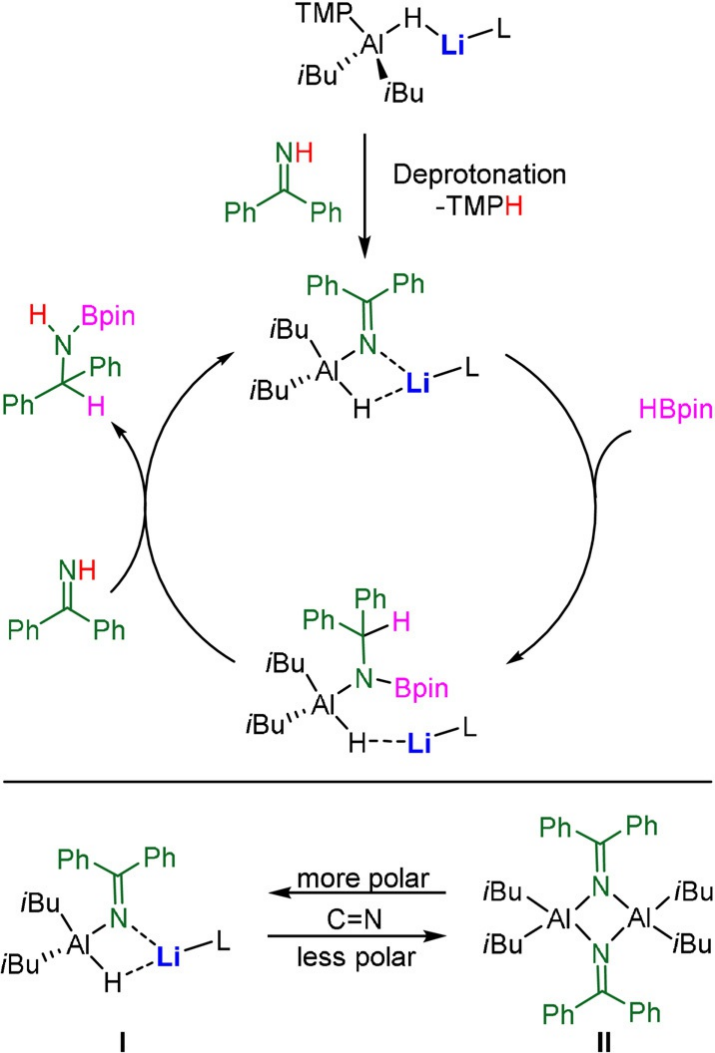
Top: Proposed catalytic cycle for the hydroboration of benzophenone imine catalysed by **4**; bottom: proposed intermediates **I** and **II** formed by catalysts **4** and **7**, respectively.

Cooperativity was also revealed between alkali metals and aluminium, as evidenced by one of the best‐known bimetallic compounds, namely lithium aluminium hydride, LiAlH_4_. Recently Harder and co‐workers used it as a catalyst for the hydrogenation of imines.^[7c]^ Our own group employed various derivatives of LiAlH_4_ for catalytic hydrophosphination^[5a]^ and hydroboration[^3g^, ^3h^, ^3i^] processes. For the latter we carried out a comparative study on the performance of neutral (monometallic) and anionic (bimetallic) aluminium complexes (depicted in Scheme 3), and found that the bimetallic catalysts ([(*i*Bu)_2_Al(TMP)(H)Li]_2_ (**4**), [(HMDS)_2_Al(H)(μ‐H)Li⋅(THF)_3_] (**5**), [(*i*Bu)_3_Al(H)Li] (**6**)) outperform their monometallic counterparts ([(*i*Bu)_2_Al(TMP)] (**7**), [(HMDS)_2_Al(H)] (**8**), [(*i*Bu)_3_Al(H)] (**9**)) in the conversion of aldehydes, ketones, and imines. We proposed that the bimetallic compounds have superior polarizing abilities than the corresponding monometallic aluminium complexes. This is exemplified in the catalytic cycle of the hydroboration of benzophenone imine by **4** and **7** shown in Scheme 4. After deprotonation of the substrate by **4**, the resulting intermediate **I** experiences a higher degree of polarization in the presence of the Lewis acidic Li (by coordination of Li to N) compared to the neutral version **II** (formed by deprotonation of benzophenone imine by **7**). Thus, the insertion of HBpin into the C=N bond proceeds more readily for the bimetallic compound **I**.^[3i]^ Harder and co‐workers reinforced this hypothesis by a DFT study in their report on LiAlH_4_‐catalysed imine hydrogenation, where they showed that the bridging Al‐H‐Li interactions are maintained throughout the catalytic cycle.[^7c^, ^7g^]

### 2.2 The Rise of Organosodium Mediation in Stoichiometric Organic Transformations

With more and more chemists and funding agencies understandably placing greater emphasis on the sustainable future of our planet, research into terrestrially abundant, inexpensive, and non‐toxic elements is gathering momentum. Sodium trumps lithium in this category, as it is the most abundant alkali metal in the Earth's crust and the sixth most abundant element overall, whereas lithium is a relatively scarce element.^[15]^ Organosodium compounds have long been inferior to organolithium compounds in terms of effectiveness and convenience across the general landscape of synthetic applications, but with sustainability now an added important consideration, some researchers are taking on the challenge of developing novel new lines of organosodium research, which in time could stimulate a massive growth in the use of organosodium reagents in organic transformations.

**Scheme 5:**
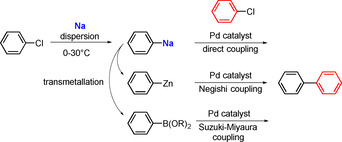
Sequential preparation of arylsodium compounds, transmetallations to arylzinc and arylboron compounds, and Pd‐catalysed C−C coupling reactions.

A particularly exciting new line of organosodium research is in the area of transition‐metal‐catalysed cross‐coupling. The mind‐set behind this research is simple: replace the less‐reactive lithium and magnesium by the more reactive sodium so that the organometallic compounds required for the transmetallation step could be prepared from the preferred halide starting materials, organic chlorides.^[16]^ These chlorides are less expensive than organic bromides and organic iodides, but having strong C−Cl bonds they are more challenging to use with lithium and magnesium and generally require harsh conditions. The use of sodium in the form of a fine dispersion led to reactions with a wide range of aryl chlorides containing different substituents (e.g. alkyl, aryl, silyl, methoxy, or dimethylamino groups) affording the desired arylsodium compounds under mild conditions in good yields with no signs (or traces) of the homocoupled biaryl side product, as reported by Asako, Nakajima, and Takai. Even sterically crowded compounds such as tri‐*t*‐butylphenylsodium could be made by this method. Since these arylsodium compounds could transfer their aryl groups to zinc (via ZnCl_2_⋅TMEDA) and boron (via MeOBpin) to form arylzinc and arylboron compounds, respectively, palladium‐catalysed Negishi and Suzuki–Miyaura cross‐coupling reactions could then be performed with a second aryl chloride by using the Pd‐PEPPSI‐IPr catalyst (Scheme 5).

**Scheme 6:**
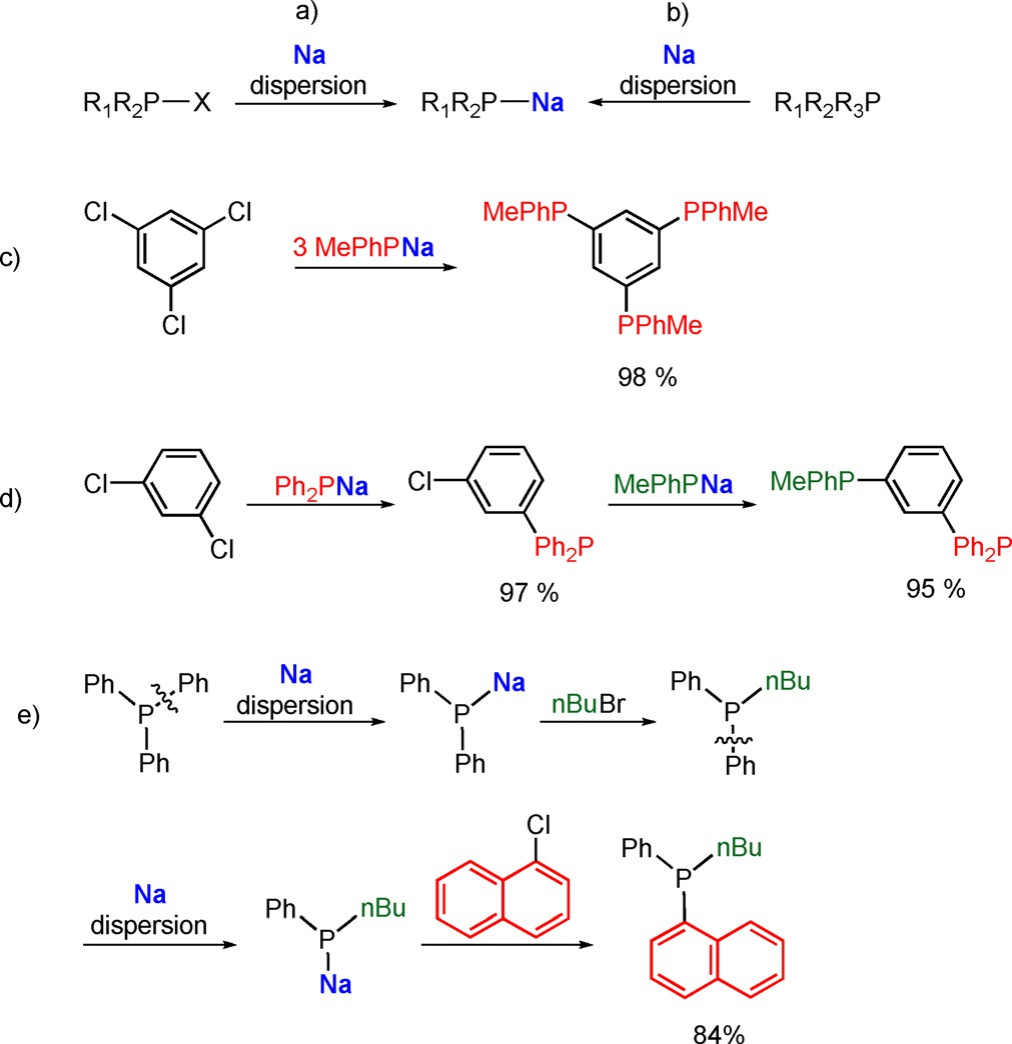
Synthesis of sodium phosphides R_1_R_2_PNa starting from a) R_1_R_2_P‐X; b) R_1_R_2_R_3_P. c) One‐pot synthesis of multidentate arylphosphines. d) One‐pot synthesis of unsymmetrical, multidentate arylphosphines. e) One‐pot sequential synthesis of an unsymmetrical, tertiary phosphine.

The Han group is also taking advantage of the clean reactivity of finely dispersed sodium for the generation of sodium phosphides R_1_R_2_PNa. These compounds can be produced in high yields and short reaction times by treatment of the sodium dispersion with either phosphine halides (R_1_R_2_P‐X; Scheme 6 a) or directly from a tertiary phosphine (R_1_R_2_R_3_P; R_1_,R_2_,R_3_=alkyl, aryl; Scheme 6 b). When different substituents are attached to the phosphine, the more electron deficient C−P bond is generally cleaved; for example, a phenyl group is preferentially cleaved over a tolyl group, or a benzonitrile substituent is more likely to be cleaved off than a phenyl group. This effective and useful method can be used for the preparation of unsymmetrical tertiary phosphines (see Scheme 6 d,e). Both procedures with Na show higher yields compared to those employing K, and are superior to those with Li, since the quenching of by‐products (e.g. phenyllithium or excess Li) is avoided. The sodium phosphides are capable of undergoing reactions with various halide substrates to form tertiary phosphines and, advantageously, they exhibit better conversions when smaller halides are attached, thus opening the door to the cheaper and readily accessible chloroarenes as well as for challenging C−F bonds. This method is straightforward for the synthesis of valuable multidentate arylphosphines, as shown in Scheme 6. Trichlorobenzene can be converted into the corresponding triphosphine in an effortless one‐pot synthesis in quantitative yields (Scheme 6 c). The substitution with different R attachments proceeds smoothly, whereby every sodium phosphide is added successively after each substitution is finished without any interim purification steps (Scheme 6 d). Whereas the synthesis of unsymmetrical tertiary phosphines is quite often a tedious procedure, using this method for tailoring the desired phosphine is a convenient one‐pot procedure (Scheme 6 e). Starting with Ph_3_P, one phenyl group can be cleaved by treatment with finely dispersed Na to access Ph_2_PNa. Subsequent addition of *n*‐BuBr gives Ph_2_(*n*Bu)P. The addition of another equivalent of finely dispersed Na gives (Ph)(*n*Bu)PNa, which ultimately is converted into the desired unsymmetrical tertiary phosphine (Ph)(*n*Bu)(1‐naphthyl)P in an overall yield of 84 % upon introducing 1‐naphthyl chloride.^[17]^

**Scheme 7:**
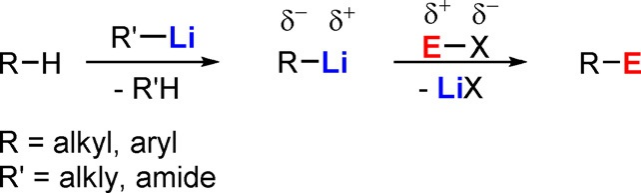
General equation for the classical two‐step metallation and electrophilic interception process.

A fundamentally important reaction long synonymous with organolithium chemistry is metallation. For example, using metallation to functionalise aromatic and heteroaromatic substrates is of industrial importance, especially for the elaboration of pharmaceuticals and agrochemicals.^[18]^ In metallation, an organolithium reagent mediates the selective functionalization of an organic substrate by converting an inert C−H bond into a C−E bond via a lithiated intermediate containing a reactive C−Li bond that can be intercepted by an electrophile “EX” (Scheme 7).

**Scheme 8:**
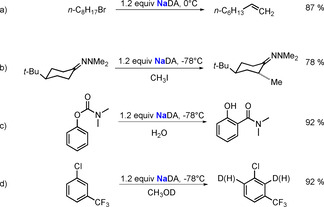
Reactions of various organic substrates with NaDA.

Lithium‐alkyl compounds, lithium amides, or mixed‐metal complexes containing Li and another alkali metal such as potassium (so called “superbases”) are usually employed for metallation applications (note that mixed‐metal superbases are not covered here, since they have been widely reviewed elsewhere).^[19]^ To militate against competitive nucleophilic addition processes with unsaturated organic substrates, sterically encumbered lithium amides are generally preferred. Equipped with two branched alkyl arms, lithium diisopropylamide (LDA) is especially suited to this role and, consequently, has been one of the most commonly utilised reagents in organic chemistry for 50 years,^[20]^ particularly in natural product syntheses.^[21]^ Although sodium diisopropylamide (NaDA) was first prepared as long ago as 1959, only a decade after its lighter sibling, it has essentially remained unused. In 2017, Collum and co‐workers summed it up by remarking that “sodium diisopropylamide has been used in about a dozen studies overall, whereas lithium diisopropylamide is probably used thousands of times daily”.^[22]^ The success of LDA is probably a factor in this unemployment, as is the generalisation that organolithium compounds tend to be orders of magnitude more stable than organosodium compounds, a fact that would also extend to the metallated intermediates formed during metallation reactions. The greater ionic character of sodium compared to that of lithium in organic environments is also a factor, since although it may enhance reactivity it can also lead to poor solubility in organic solvents or to decomposition of the solvent through its greater basicity. By the judicious choice of the solvent, namely using *N*,*N*‐dimethylethylamine (DMEA), NaDA has belatedly begun to find application.^[23]^ Prepared by mixing a sodium dispersion in toluene with isoprene and diisopropylamine in DMEA,^[24]^ NaDA was studied by Collum and co‐workers in metallation reactions of arenes, epoxides, ketones, hydrazones, dienes, alkyl halides, and vinyl halides. Surprisingly, in most cases NaDA/DMEA shows high reactivities and chemoselectivities on a par with those of the commonly utilised LDA/THF. Examples where NaDA mediation is even advantageous include the clean elimination of 1‐bromooctane to give the alkene (Scheme 8 a; LDA effects a mixture of elimination and substitution); the faster metallation of hydrazone (Scheme 8 b; the reaction with LDA exhibits a higher axial selectivity, although is significantly slower); the rapid *ortho* metallation of a carbamate in contrast to the sluggish performance of LDA/THF (Scheme 8 c); and the metallation of 3‐trifluoromethylphenyl chloride to give the 2‐sodiated intermediate at −78 °C, in contrast to a mixture of 2‐ and 6‐lithiated intermediates with LDA/THF (Scheme 8 d).

**Scheme 9:**
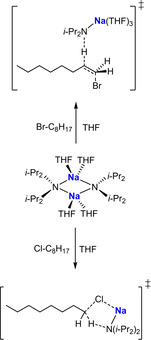
Transition states for the sodiation of bromooctane (top) and chlorooctane (bottom) with NaDA.

A follow‐up kinetic study on the NaDA‐mediated metallation of an assortment of alkyl halides in THF/hexane or THF/DMEA revealed that 1‐halooctanes (chloro, bromo, or iodo) undergo smooth and efficient elimination of sodium halide as opposed to a mixture of elimination and substitution as often observed with LDA.^[25]^ Various mechanisms have been proposed, although all involve solvated variations of the NaDA monomer. For example, with 1‐bromooctane, an E2‐like elimination pathway via a trisolvated monomer is implied (Scheme 9, top); whereas this switches with 1‐chlorooctane to a disolvated monomer through a carbenoid mechanism (Scheme 9, bottom). On the negative side, NaDA mediation failed with *n*‐alkyl fluorides because of competitive metallation and subsequent decomposition of the THF solvent. NaDA‐mediated deprotonations of 1,3‐dimethoxybenzene and related methoxylated arenes also show exclusively monomer‐based mechanisms with two or three coordinated THF ligands.^[26]^

**Scheme 10:**
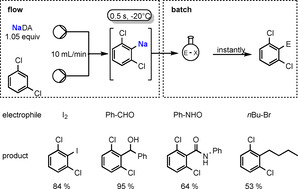
Sodiation of 1,3‐dichlorobenzene using a microflow reactor. Quenching of the sodium intermediate with various assorted electrophiles using an alternative batch method gives functionalized dichlorobenzenes.

NaDA in DMEA has also proved efficient for the sodiation of (hetero)arenes in continuous microflow reactors (Scheme 10).^[27]^ Establishing the optimal conditions to be a flow rate of 10 mL min^−1^, the Knochel group found that complete sodiation of the test reagent, 1,3‐dichlorobenzene, could be accomplished within 0.5 seconds at −20 °C; whereas under conventional batch conditions this sodiation would need to be conducted at −78 °C to avoid decomposition processes. The resulting 2,6‐dichlorophenylsodium can be intercepted immediately in batch reactions with a range of electrophiles.

**Scheme 11:**
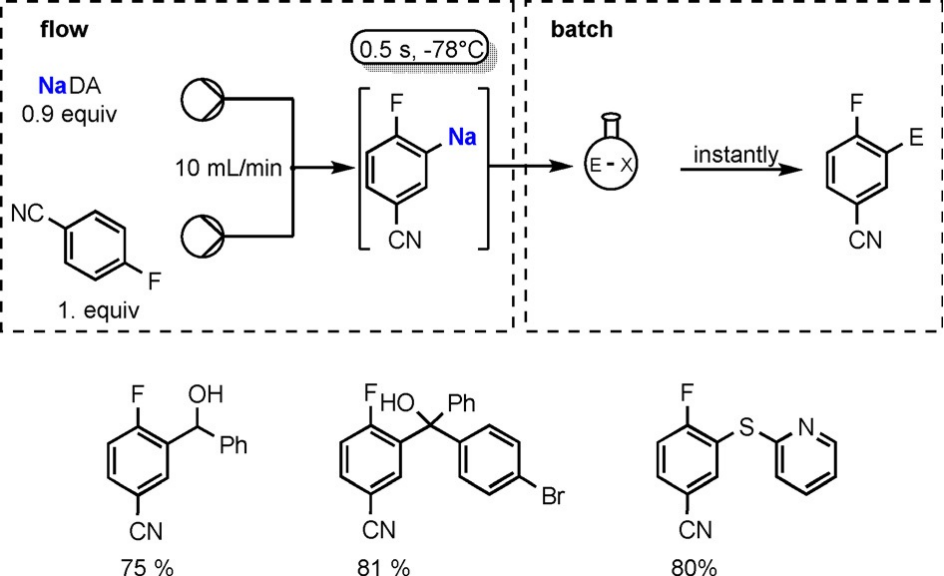
Sodiation of 4‐fluorobenzonitrile (flow). Quenching of sodium intermediate with different electrophiles (batch) gives functionalized benzonitriles.

This flow sodiation is successful with an impressive variety of organic substrates including haloarenes and halo(hetero)arenes, many of which are highly sensitive and decompose when sodiation is attempted under conventional batch conditions. 4‐Fluorobenzonitrile is such an example, as usually the nitrile substituent would be attacked using conventional sodiation, but under continuous‐flow conditions it is sodiated *ortho* to the F substituent. The subsequent batch treatment of this sodium intermediate with an aldehyde, a ketone, or a disulfide leads to the desired polyfunctionalised benzonitrile in high yield (Scheme 11).

**Scheme 12:**
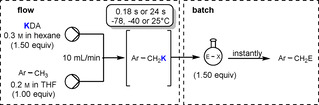
Metallation of methyl‐substituted (hetero)arenes by using KDA/TMEDA in continuous flow (flow). Quenching of the potassium intermediate with electrophiles (batch) gives access to functionalized methyl‐substituted (hetero)arenes.

As is the case with NaDA, a new preparative redox method for making potassium diisopropylamide, KDA, that is free from lithium impurities has enhanced its synthetic mediation qualities.^[28]^ Accessed from a 3:1:1:0.5 mixture of potassium, diisopropylamine, TMEDA, and isoprene, this new form of KDA, used in situ as the solvate KDA(TMEDA),^[29]^ has also been utilised in commercial microflow reactors for the potassiation of arenes and (hetero)arenes between −78 °C and 25 °C with reaction times between 0.2 s and 24 s and a combined flow rate of 10 mL min^−1^. With these substrates, potassium–hydrogen exchange occurs selectively on the aryl ring, and the potassiated intermediates can then undergo immediate electrophilic interception with, for example, aldehydes, ketones, alkyl and allylic halides, and disulfides, to produce the target functionalized (hetero)arenes in high yields. The scope of these KDA(TMEDA)‐mediated reactions is extended to the lateral potassiation of methyl‐substituted (hetero)arenes to generate, in turn, benzylic potassium intermediates and methyl‐functionalised heteroarenes, as illustrated with toluene in Scheme 12.

Renewed interest in organosodium chemistry and its comparison with organolithium chemistry have not been limited to the diisopropylamide ligand. The sodium congener of another utility amide, 2,2,6,6‐tetramethylpiperide, TMP, is also being investigated. Since its preparation in 1999,^[30]^ NaTMP has mainly been of interest in the development of synergistic bimetallic chemistry. However, Takai and co‐workers have recently reported examples where NaTMP, made by sodium dispersion techniques to ensure no lithium contamination is present, proves superior in terms of reactivity and selectivity compared to its lighter sibling LiTMP in Brønsted base applications for organic synthesis.^[31]^ This includes the stereoselective Wittig reaction, where deprotonation of the phosphonium salt by LiTMP or NaTMP in THF/hexane and the subsequent reaction with 2‐naphthaldehyde gave similar high yields of the alkene product (both 88 %) but markedly different *E*/*Z* ratios (57:43 or 7:93; Scheme 13 a).

**Scheme 13:**
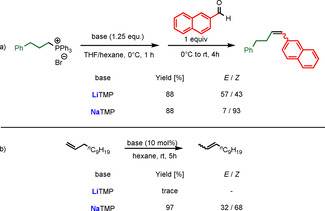
a) Wittig reaction carried out with LiTMP and NaTMP. b) Li/NaTMP‐catalysed isomerization of 1‐dodecene.

NaTMP also outperformed LiTMP in the terminal‐to‐internal double‐bond isomerization reactions of 1‐dodecene (Scheme 13 b). Whereas, LiTMP failed to catalyse the isomerization reaction in hexane at ambient temperature, NaTMP transformed 1‐dodecene into 2‐dodecene as a mixture of *E*/*Z* stereoisomers, thus reflecting its higher Brønsted basicity over that of LiTMP. This same study demonstrated that NaTMP could deprotonate the heteroarenes benzofuran, benzothiophene, and dibenzofuran under mild conditions to produce sodium intermediates in situ that were stable enough to be intercepted with assorted electrophiles.

**Figure 3:**
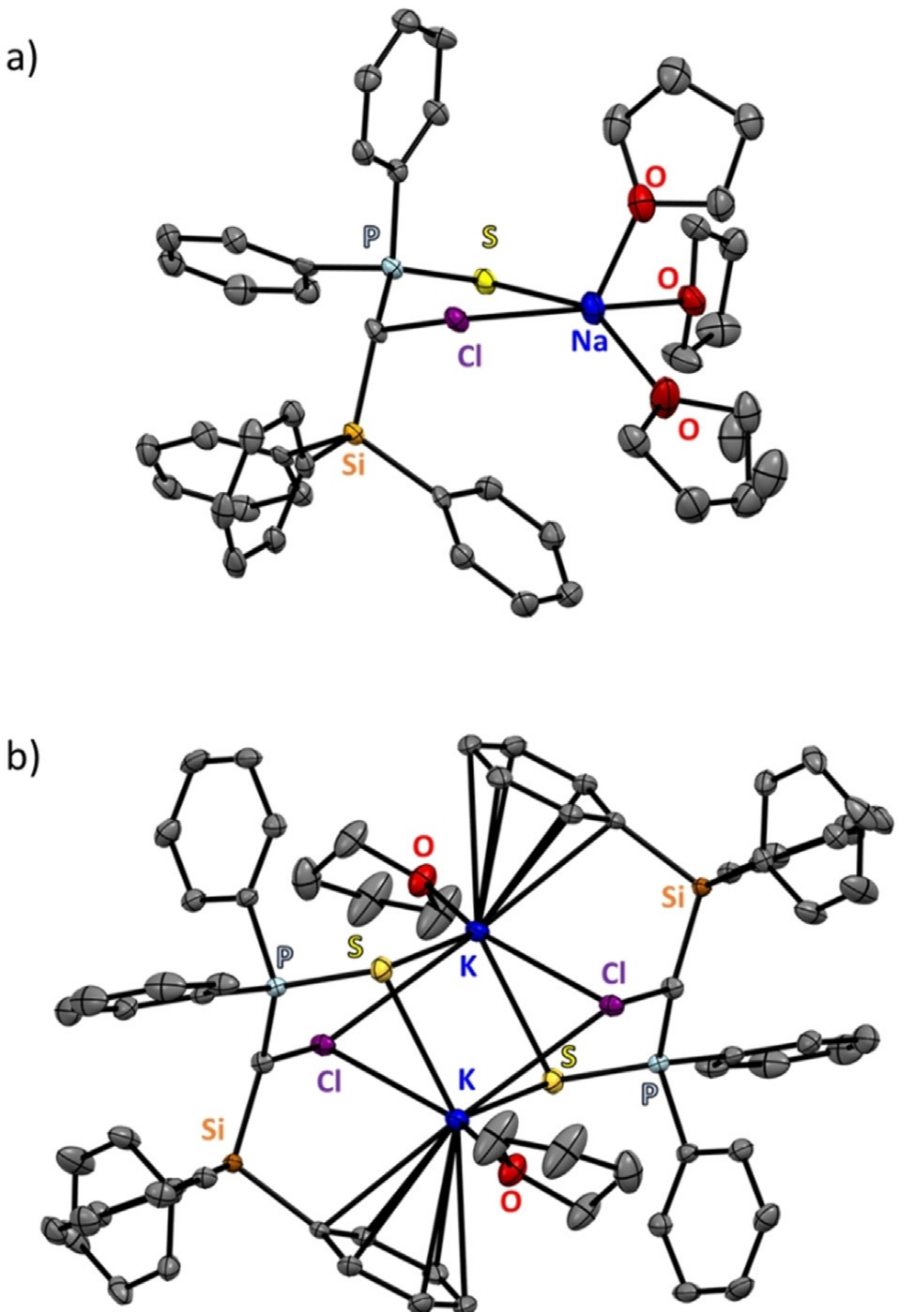
Molecular structures of a) sodium carbenoid and b) potassium carbenoid. Hydrogen atoms are omitted for clarity.

As mentioned earlier, the stability of alkali‐metal reagents usually decreases on descending down the group. Intriguingly, however, the opposite trend was observed by the Gessner group, whose research focuses on the synthesis of alkali‐metal carbenoids.^[32]^ In 2016 they reported a series of alkali‐metal carbenoids, with the sodium and potassium congeners being the first structurally characterised complexes of this compound class (carbenoids are usually reactive intermediates in several reactions, for example, the Simmons–Smith reaction, and incorporate a halogen next to the carbene unit). Thus, the decomposition of alkali‐metal carbenoids is driven by the formation and precipitation of the metal halide. Gessner and co‐workers found that, whereas the lithium complex undergoes decomposition even at 0 °C, the heavier alkali metal sodium and potassium congeners were surprisingly stable up to 30 °C. This superior stability is credited to the diminished Lewis acidity of the heavier alkali metals combined with the greater polarity of the M–C interactions compared to those of lithium. The latter effect decreases the polarization of the carbenoid C−Cl bond and concomitantly impedes the elimination of MCl. X‐ray diffraction studies (the molecular structure of the potassium carboenoid is depicted in Figure 3) indeed revealed a significant weakening of the C−Cl bond, as evidenced by its elongation compared to the bond in the precursor (by 0.05 Å for Na, 0.03 Å for K).^[32b]^

### 2.3 Low‐Valent Aluminium Chemistry

**Scheme 14:**
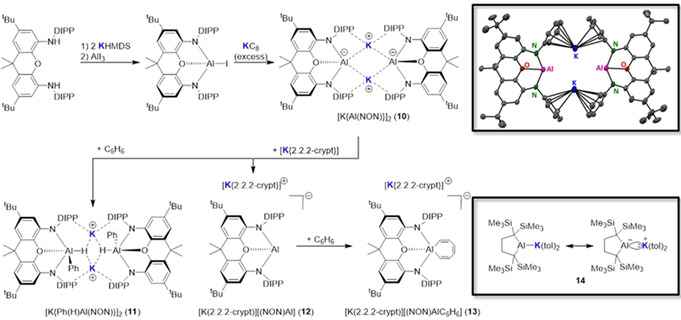
Synthesis of complexes **10**–**13**; solid‐state structure of **10** and resonance structures of **14**.

Potassium is making a significant impact in the development of low‐valent aluminium chemistry.[^33^, ^34^, ^35^, ^36^, ^37^, ^38^, ^39^, ^40^, ^41^] This is particularly evident in the first report of an anionic aluminium nucleophile, in the dimethylxanthine‐supported potassium aluminyl [K{Al(NON)}]_2_ (**10**, NON=4,5‐bis(2,6‐diisopropylanilido)‐2,7‐di‐*tert*‐butyl‐9,9‐dimethylxanthene) by Goicoechea, Aldridge, and co‐workers.^[33]^ Potassium plays a threefold role, namely in the synthesis, the structure, and the reactivity of **10**. The nucleophilic aluminyl anion is generated in a three‐step process (Scheme 14). Double deprotonation of the secondary aniline (NON)H_2_ with KHMDS and then treatment with AlI_3_ generates the Al^III^ iodide (NON)AlI, which, in turn, is reduced by excess potassium graphite to form **10**. The molecular structure of crystalline **10** (Scheme 14) shows it to be a centrosymmetric dimer, composed of two formally anionic [Al(NON)]^−^ units, where dimerization is effected by flanking potassium⋅⋅⋅π‐arene interactions. Support for the importance of these bridging interactions to the stability of the dimeric structure was provided by DOSY NMR measurements, which suggest that the dimer is retained in aromatic solvents. Despite its low Al valency, aluminyl **10** is stable for several days at 300 K in benzene solution, but interestingly on raising the temperature by 30 K, it converts into the Al^III^ species [K{Ph(H)Al(NON)}]_2_ (**11**) through formal oxidative cleavage of a C−H bond of benzene and concomitant oxidative addition at Al^I^. This novel reaction again illustrates the importance of potassium, as it holds the dimer **11** together through interactions with the DIPP (diisopropylphenyl) groups and the emergent hydrides (Scheme 14). That said, the addition of [2.2.2]cryptand (4,7,13,16,21,24‐hexaoxa‐1,10‐diazabicyclo[8.8.8]hexacosane) to aluminyl **10** generates monomeric [K(2.2.2‐crypt)][(NON)Al] (**12**), where the K centre is sequestered from the anionic moiety.^[34]^ This release of the stabilising K cation modifies the reactivity of the anionic moiety, leading not to C−H bond activation but rather to C−C bond activation and ring‐opening of benzene to form a seven‐membered AlC_6_H_6_ metallacycle in [K(2.2.2‐crypt)][(NON)AlC_6_H_6_] (**13**; Scheme 14). In contrast to the charge‐separated monomer **12**, when the dianionic moiety is a tetrakis(trimethylsilyl)butylene ligand, a contacted ion‐pair monomer **14** is formed, where the potassium engages directly with the Al centre in the shortest K–Al length reported to date [3.4549(5) Å], with the remainder of the potassium coordination sphere made up of π–arene interactions with two toluene molecules.^[35]^

**Scheme 15:**
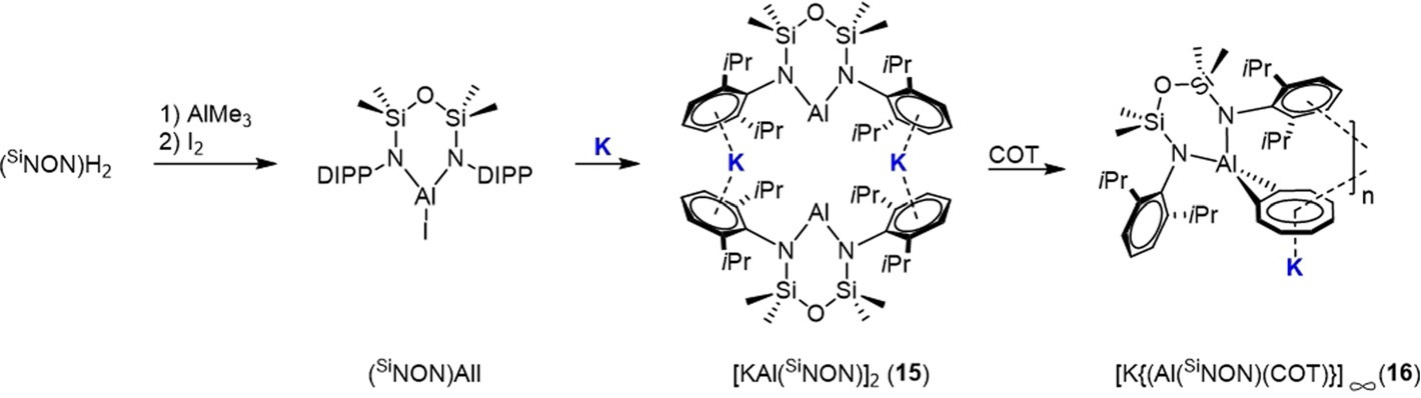
Synthesis of (^Si^NON)AlI, [KAl(^Si^NON)]_2_ (**15**) and [K{Al(^Si^NON)(COT)}]_∞_ (**16**).

Elemental potassium can also be used as an alternative source to generate the related potassium aluminyl complex [KAl(^Si^NON)]_2_ (**15**; ^Si^NON={O(SiMe_2_N^Ar^)_2_}^2−^, Ar=2,6‐iPr_2_C_6_H_3_) in a redox reaction with (^Si^NON)AlI (Scheme 15) as established by the Coles group.^[36]^ Potassium⋅⋅⋅π‐arene interactions are also a central feature of the dimeric structure of **14,** with the two K centres acting as bridges between two aluminyl anions. The reaction of **15** with 1,3,5,7‐cyclooctatetraene (COT) leads to unsolvated (in the Lewis base sense) inverse sandwich complex [K{Al(^Si^NON)(COT)}]_∞_ (**16**), where the COT ligand engages in a μ^2^‐η^2^:η^8^ coordination with the Al(^Si^NON) anion and K cation, respectively. The large size and “soft” bonding character of the K cation also enables intermolecular interactions with a neighbouring COT ligand (η^3^‐) and Ar substituent (η^5^‐), which propagates the structure into a helical polymer (Scheme 15).

**Scheme 16:**
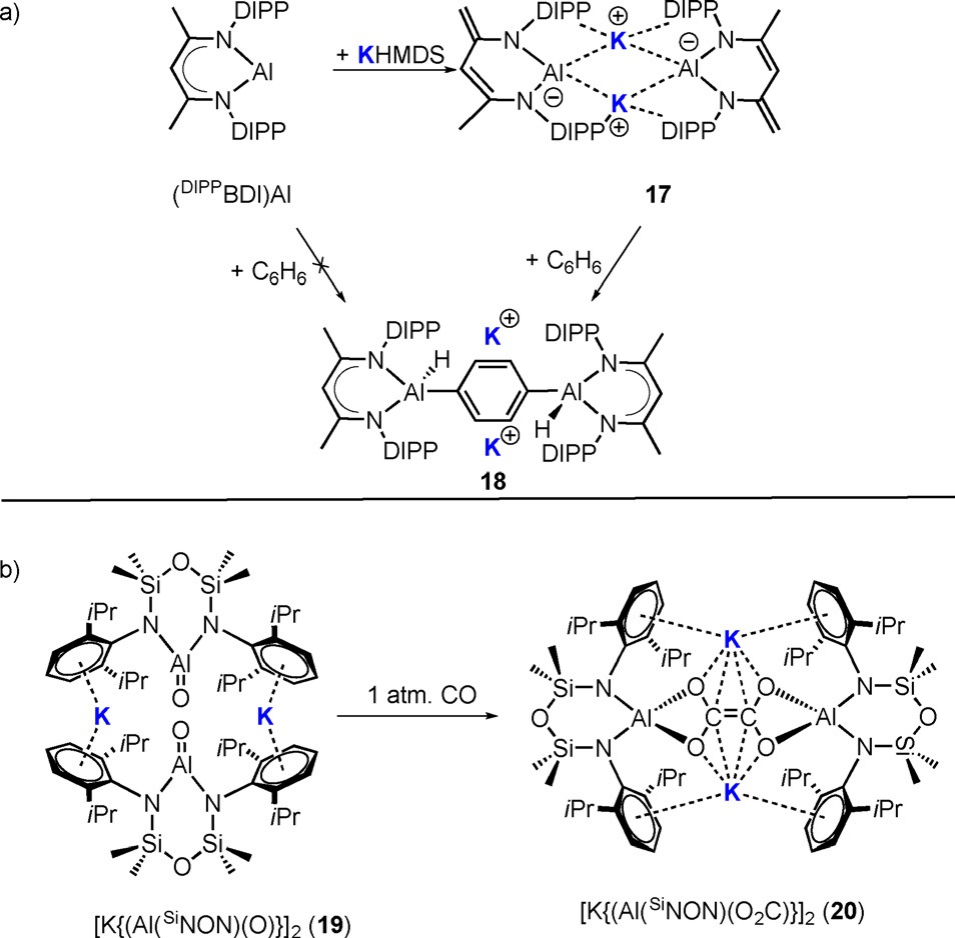
Synergistic reactions between K and Al: a) (^DIPP^BDI)Al reduces in the presence of KHMDS/benzene at the 1,4‐positions. b) Reaction of aluminoxane [K{Al(NON^Dipp^)(O)}]_2_ (**19**) with 1 atm CO.

Noting that all literature anionic Al complexes have to date been electronically balanced by K^+^ cations, Harder and co‐workers added the potassium reagent KHMDS to the neutral low valent compound (^DIPP^BDI)Al (BDI=β‐diketiminate).^[37]^ Surprisingly, the expected nucleophilic addition reaction did not occur, but instead deprotonation took place at a backbone Me group in the ^DIPP^BDI ligand to generate the dianionic bisamide [H_2_C=C(NAr)‐C(H)=C(Me)‐NAr] **17**, with the by‐product presumably HMDS(H). Crystallographic characterisation of **17** revealed a dimeric arrangement akin to that of **10**, in which formally (Al)^−^ anions are bridged by K^+^ cations that once again exhibit strong K^+^⋅⋅⋅π‐arene interactions with the DIPP groups. The mediation of the reactivity of potassium is clearly evident in the reaction of **17** with benzene (Scheme 16 a). In the absence of the potassium reagent, (^DIPP^BDI)Al is inert to benzene, but **17** does react and in a remarkable way, inducing twofold C−H bond activation of benzene with its *para*‐phenylene unit trapped between the two Al centres, which each carry a released hydride ion in the product **18**. One K^+^ ion sits over the phenylene bridge, engaging in interactions with the DIPP π‐systems, while the second K^+^ ion propagates the coordination polymer by bridging through K^+^⋅⋅⋅BDI and K^+^⋅⋅⋅H(Al) contacts. This **17** to **18** transformation has been likened to the ring‐template‐controlled double metallation reactions of arenes found in inverse crown chemistry.

The authors strongly advocated the alkali‐metal mediation theme that is the focus of this Review, and stressed, on the basis of DFT calculations, that it is important to take into account the role of the counter cation K^+^, as its presence or absence can markedly alter the outcomes of anionic Al reactions, which appear to have a synergistic mixed‐metal (K/Al) origin. Coles and co‐workers witnessed related K/Al synergistic reactivity in the production of the ethenetetraolate [K{Al(^Si^NON)(O_2_C)}]_2_ (**20**) on exposing carbon monoxide to the potassium aluminoxane [K{Al(^Si^NON)(O)}]_2_ (**19**; Scheme 16 b).^[38]^

### 2.4 Polymerization

**Figure 4:**
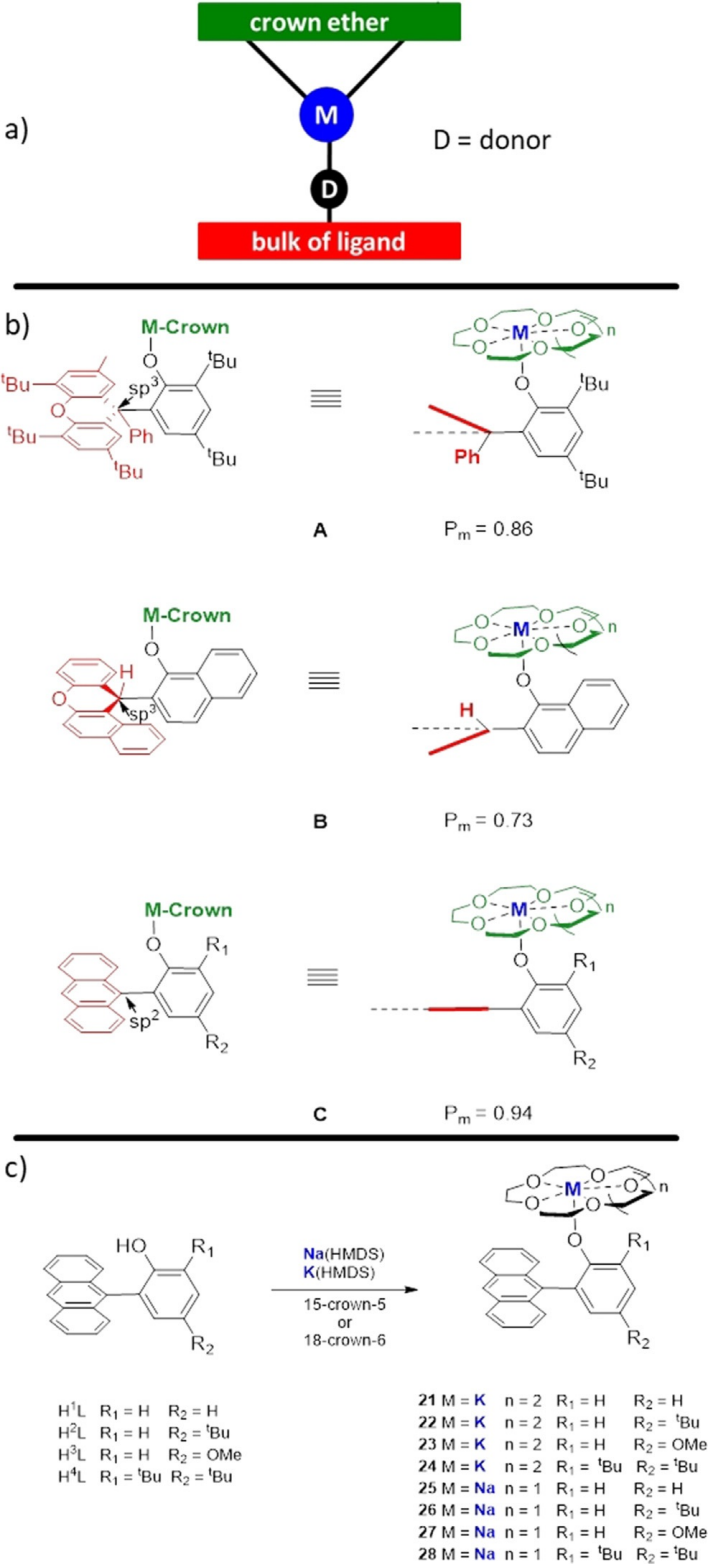
a) General design of an alkali‐metal‐based catalyst for ROP of lactide; b) **A**: complex where the substituent in the *ortho* position points towards the metal centre; **B**: complex where the substituent in the *ortho* position points away from the metal centre; **C**: complex where the substituent in the *ortho* position and crown ether confine the metal centre perfectly. c) Synthesis of complexes **21**–**28**.

Another important research area where alkali‐metal catalysts are frequently applied is in the polymerization of lactide. Polylactide is non‐toxic, biodegradable, and biocompatible. It is used widely in fields such as agriculture, packaging, food, and medicine. Considering that alkali metals are non‐toxic and found in the human body (especially Na and K), catalysts based on these innocuous metals are very attractive, since traces of the catalyst used can remain in the polymer; depending on which metal is present, this can potentially lead to harmful effects. Within the past two decades several suitable complexes for the ring‐opening polymerization of lactide have been reported and some general trends have been observed, such as the reactivity of the catalysts increases on descending the first main group from Li to Na to K.[^42^, ^43^, ^44^, ^45^, ^46^, ^47^, ^48^, ^49^, ^50^, ^51^, ^52^, ^53^] This is attributed mainly to the larger size of the metal facilitating coordination of the substrate. However, the opposite trend is observed with regard to selectivity, as it decreases from Li to Na to K.[^50^, ^51^, ^54^] The lower Lewis acidity and the resulting weaker bond to the substrate can prevent the growth of long and defined polymeric chains. To overcome this drawback, a general catalyst design has been developed, which is depicted in Figure 4 a. The idea is to sandwich the catalytically active centre between the two planes, thereby embedding the cation in a defined space to boost the interaction of the monomer and the active end of the polymer chain. One side is confined by a bulky ligand, which forms an ionic bond to the metal. Typically applied ligand classes incorporate oxygen[^42^, ^43^, ^44^, ^45^, ^46^, ^47^, ^48^, ^53^, ^55^, ^56^, ^57^, ^58^, ^59^, ^60^] or nitrogen donors,[^49^, ^61^] or a combination of both types.[^50^, ^51^, ^54^, ^62^, ^63^, ^64^] Catalysts with electron richer ligands showed higher catalytic activity;[^62^, ^63^] whereas more steric bulk around the metal had a detrimental influence on the performance.^[46]^ The other side of the metal's coordination sphere is usually restricted by a crown ether. This leads to the benefit that the complexes are broken down to monomers, which then results in a boost in the reactivity as there are more active centres available. The size of the donor has to be chosen carefully, since a too‐crowded cation shows less catalytic activity.[^42^, ^47^, ^48^, ^62^] Wu et al. synthesized a series of complexes (depicted in Figure 4 b) and the importance of a well‐defined space around the metal was evaluated. All compounds were synthesized by simple deprotonation of the phenol with KHMDS or NaHMDS and subsequent addition of the corresponding crown ether. In complexes **A** ^[46]^ and **B**,^[47]^ a xanthyl group is attached to the phenolate in the *ortho* position. The plane can be bent up (**A**) or down (**B**) at the sp^3^‐hybridized carbon atom, which results in a flexible and ill‐defined area around the metal. Substitution of this residue by an anthryl group (**C**)^[48]^ gives a more rigid and confined space. As a result, **C** shows the highest *P*_m_ value of 0.94 (**A**=0.86, **B**=0.79; the *P*_m_ value describes the isotacticity in the polymer). A series of Na and K complexes of **C** with different substituents on the phenolate (see Figure 4 c) were tested in the ROP of lactide with benzyl alcohol (BnOH) as a co‐initiator; a selection of results are listed in Table 1. The K complexes show an increase in activity from **23** to **22** to **21**, which indicates that the electron‐donating group accelerates the reaction (entries 1–3). Therefore, a higher electron density on the phenoxy oxygen atom enhances reactivity. The same trend was observed for the Na complexes (entries 5–8), although they are slightly less active (since the Na−O bond is less ionic than the K−O bond). Adding steric bulk to the *ortho* position (R_1_=*t*‐Bu) decreased the reactivity for K (entry 4) and Na (entry 8) because the active centre becomes too crowded. Changing the solvent from toluene to THF resulted in a decrease in reactivity, since there is a competing coordination of THF and lactide towards the K^+^ cation (entry 9). Lowering the temperature to −70 °C gave a more controlled reaction, and the best isoselectivity (*P*_m_=0.94) was observed for complex **21** (entry 10). Additionally, the obtained molecular‐weight distributions show that the polymerizations catalysed by **21**–**28** are living.^[48]^

**Table 1:** Selected data to show the performance of the *rac*‐lactide polymerization catalysed by **21**–**28**.

Entry	Cat.	[Cat.]_0_/[*rac*‐La]_0_/[BnOH]_0_	*t* [min]	Conversion [%]	*M*_n,obs_ [g mol^−1^]	*M*_n,calc_ [g mol^−1^]	*P*_m_	*T* [°C]	Solvent
1	**21**	1:100:1	2	93	12 800	13 500	0.72	RT	toluene
2	**22**	1:100:1	2	99	11 600	14 400	0.73	RT	toluene
3	**23**	1:100:1	1.5	99	13 500	14 400	0.65	RT	toluene
4	**24**	1:100:1	5	99	14 400	14 400	0.72	RT	toluene
5	**25**	1:100:1	3	99	17 200	14 400	0.72	RT	toluene
6	**26**	1:100:1	2	85	12 700	12 300	0.67	RT	toluene
7	**27**	1:100:1	2	92	13 300	13 400	0.66	RT	toluene
8	**28**	1:100:1	5	99	14 100	14 400	0.67	RT	toluene
9	**21**	1:100:1	30	68	7200	9900	0.70	RT	THF
10	**21**	1:200:1	16	93	26 500	26 900	0.94	−70	toluene

## 3 Organolithium Chemistry: Green Shoots of a New Future

The content of this Review could leave the impression that the glory days of organolithium chemistry have long passed. However, like a true champion, it continues to adapt and diversify to meet the challenging demands of today's world. Remarkably, in recent times, significant progress has been made even with regard to the Achilles’ heel of organolithium chemistry, namely its inability to operate in the presence of air and/or moisture. A century ago, the aforementioned Wilhelm Schlenk designed his special inert‐atmosphere glassware to protect organolithium compounds from these nemeses, which, on the merest contact, will rapidly cause decomposition of “RLi” to more thermodynamically stable Li−O bonded and covalent R‐H products. Taking on the seemingly “impossible” challenge of finding a way to perform organolithium chemistry under aerobic hydrous conditions has been overlain with a drive towards creating a more sustainable “Green” chemistry world.^[65]^ In this context, this means predominately replacing hazardous organic reaction solvents by safe, green, and bio‐renewable reaction media that are not based on crude petroleum. Three leading authorities—Capriati, Garcia‐Alvarez, and Hevia—have recently surveyed this emerging field in their article “the future of polar organometallic chemistry written in bio‐based solvents and water”.^[66]^

**Scheme 17:**
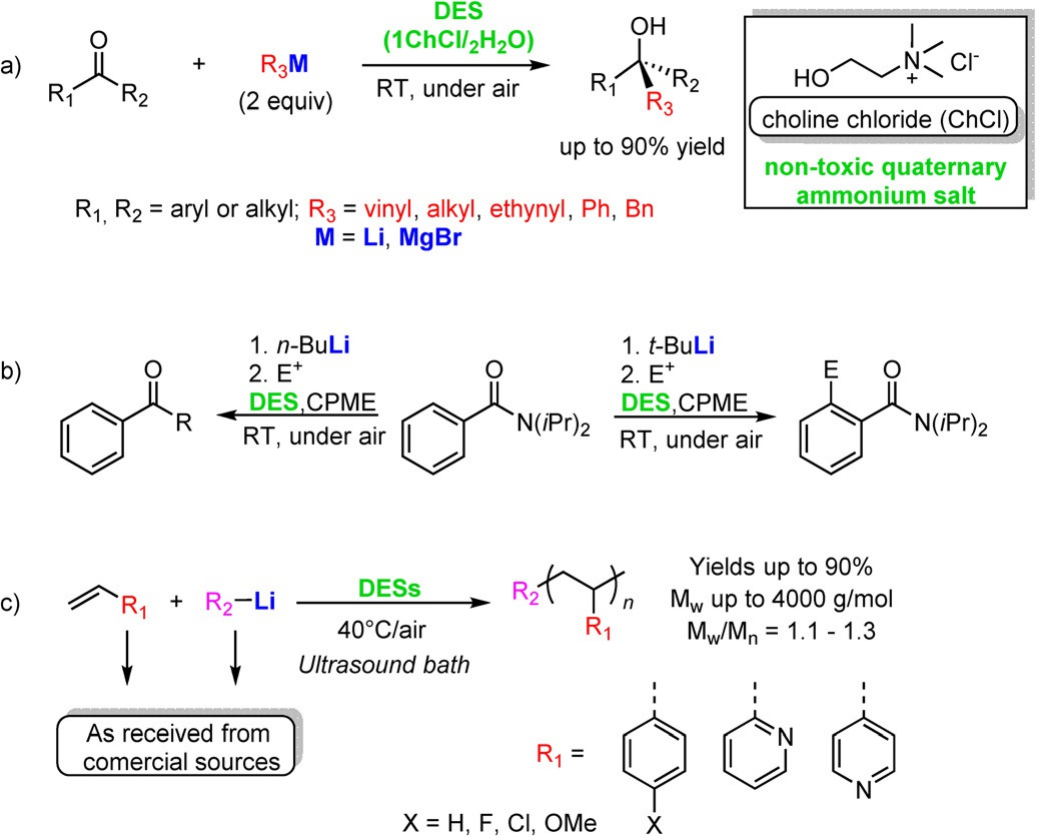
Representative reactions in ethereal/eutectic mixtures in the presence of air and moisture: a) nucleophilic additions of organometallic reagents to ketones, b) *ortho*‐lithiation and nucleophilic acyl substitution of a benzamide derivative, and c) organolithium‐promoted anionic polymerization of olefins.

Organolithium compounds unintentionally featuring water as a ligand have appeared infrequently in the literature, such as 2‐mercaptobenzoxazolyl(TMEDA)lithium monohydrate, reported by Snaith, Wright, and co‐workers in 1990.^[67]^ Today, examples can be found where organolithium reactions are carried out intentionally in the presence of water. Hevia, García‐Álvarez, and co‐workers reported the chemoselective alkylation of alkyl and aryl ketones by both RLi and RMgX (Grignard) polar reagents to generate tertiary alcohols (Scheme 17 a).^[68]^ Water was present in the bulk DES solvent (DES=deep eutectic solvent, which are eutectic mixtures made from green, nature‐inspired components that form hydrogen‐bond networks). Remarkably, enhanced yields and superior selectivities were obtained under wet, aerobic conditions in the DES (a 1:2 choline chloride/water mixture) compared to those using a standard inert atmosphere procedure.

Nucleophilic addition is not the only organolithium reaction type to undergo this seismic change in its practice. The Capriati group has successfully extended it to the *ortho*‐lithiation of a series of *N*,*N*‐diisopropylbenzamides by employing a cyclopentyl methyl ether/DES (1:2 choline chloride/glycerol) mixture at room temperature under air.^[69]^ Electrophilic quenching after just two seconds by benzaldehyde, DMF, and assorted halogenating, silylating, and sulfurylating agents readily intercepted the carbanion formed by subjecting the amide to *t*‐butyllithium. Adding to the interest of these reactions, switching the organolithium reagent from *t*‐butyllithium to *n*‐butyllithium led to no *ortho*‐lithiation taking place but instead resulted in nucleophilic acyl substitutions (S_N_Ac) that produced mainly ketones and to a small extent tertiary alcohols from over‐addition (Scheme 17 b).

In exploiting DESs for anionic olefin polymerization, the same group have established an unprecedented air and moisture‐compatible method that utilises organolithium reagents as initiators to access a variety of polystyrenes and polyvinylpyridines (Scheme 17 c).^[70]^ Sonication significantly accelerated the reactions, which were run in a DES comprising choline chloride and glycerol in a 1:2 stoichiometry, thereby producing polymers in high yields and with low polydispersities. Since organolithium‐mediated reactions of this type can be strongly influenced by the volume and surface of the organic droplets formed by the glycerol‐ or water‐insoluble reagents present, these have been labelled as “on‐glycerol” or “on‐water” reactions, hence the use of sonication.^[71]^

## 4 Summary and Outlook

In this Review we have tried to paint a picture of the increasing utilisation of organoalkali‐metal compounds in mediating new chemistry for a diverse range of applications. Although the alkali metals often seem to be essential for the success of the applications described, their role as more of a secondary supporting nature can sometimes be glossed over. This understated appreciation contrasts with the wide acclaim given to other species used to support emerging aspects of synthesis and catalysis, such as popular sterically demanding tunable ligands (for example, NHCs and β‐diketiminates). What may be surprising to those familiar with organoalkali‐metal chemistry is that lithium is far from the centrepiece of this picture, as sodium and potassium are outperforming their lighter sibling in an increasing number of cases. This is a relatively new phenomenon, although in the bimetallic world of inverse crown chemistry, potassium and especially sodium have been in the foreground of advances in template metalation, as most recently highlighted in the regioselective tetrazincation of ferrocene mediated by sodium.^[72]^ For brevity, we have limited this Review to mediations taking place in main‐group chemistry, but exciting developments are also being realised in alkali‐metal‐mediated transition‐metal chemistry. For reasons of sustainability, Earth‐abundant metals such as iron are attracting lots of activity. The potential of alkali‐metal mediation in this arena is emphatically demonstrated in the reduction of dinitrogen and its hydrogenation to ammonia by a well‐defined potassium‐iron complex, as reported by the Holland group.^[73a]^ More recently, the same group showed that a mixed sodium‐iron complex can functionalise dinitrogen through coupling reactions with hydrocarbons.^[73b]^ A review on this related theme is likely to follow in the future.

It is hoped that the snapshots presented herein will help promote alkali‐metal mediation as a concept in its own right and prompt the recruitment of many more chemists to the area. The canvas it offers for future research lines is vast. The origins of alkali‐metal mediation are still not well understood in general, although there have been some excellent rigorous studies on a few specific systems. To date, there has been a paucity of kinetic studies and theoretical calculations. Given the often complicated nature of the aggregated structures involved in alkali‐metal chemistry, such in‐depth studies are vital to uncover transition states and map reaction profiles. More studies are also necessary in which the whole alkali‐metal series is considered. Rubidium and caesium have hardly been considered at all in alkali‐metal mediation, yet their propensity for engaging in π–arene interactions suggests they could make a significant impact in applications, such as low‐valent metal chemistry and homogeneous catalysis. The synergistic effects that underpin the special chemistry of the inverse crown bimetallic complexes have mainly involved sodium mediation, and the impression at present is that the larger coordination spheres of the heavier alkali metals would make them ideal partners for constructing useful architectures with metals such as magnesium, zinc, and aluminium. Finally, the old master lithium is far from exhausted. In fact, the recent reports of organolithium reactions taking place under aerobic and hydrous conditions represent one of the most remarkable breakthroughs in chemistry in recent times. We wait with eager anticipation to see how much this green chemistry will develop in the future.

## Conflict of interest

The authors declare no conflict of interest.

## Biographical Information

*Thomas Xaver Gentner was born in 1989 in Weiβenburg*, *Germany. He obtained his master's degree as well as his PhD at the Friedrich‐Alexander University of Erlangen‐Nuremberg under the supervision of Prof. Harder, where he undertook studies on early main‐group chemistry with a special focus on low oxidation state complexes of the alkaline‐earth metals. In 2019 he then moved to the University of Strathclyde, where he is working as a postdoctoral researcher together with Prof. Mulvey on bimetallic catalyses and alkali‐metal mediation chemistry*.

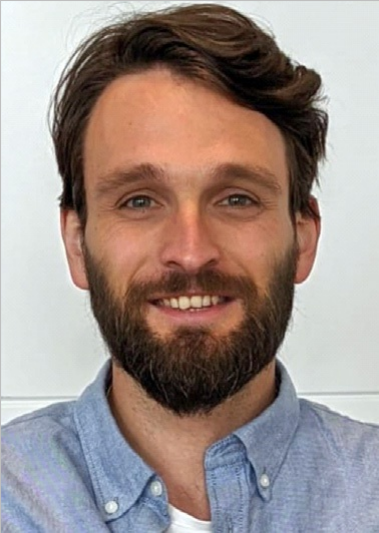

## Biographical Information

*Robert Emmet Mulvey was born in Glasgow, Scotland, and earned his BSc and PhD at the University of Strathclyde (supervisor R. Snaith). Following working as a research associate at the University of Durham (supervisor K. Wade), he returned to Strathclyde in 1986 as a Royal Society Research Fellow. He was promoted to Professor in 1995 and currently holds the 1919 chair of inorganic chemistry. He is fascinated by alkali metals and their synergistic phenomena*.